# Psychological Profile in Children and Adolescents with Severe Course Juvenile Idiopathic Arthritis

**DOI:** 10.1100/2012/841375

**Published:** 2012-05-03

**Authors:** Emanuela Russo, E. Trevisi, F. Zulian, M. A. Battaglia, D. Viel, D. Facchin, A. Chiusso, A. Martinuzzi

**Affiliations:** ^1^E. Medea Scientific Institute, Conegliano Research Centre, Via Costa Alta 37, 31015 Conegliano, Italy; ^2^Rheumatology Unit, Department of Pediatrics, University of Padua, 35128 Padova, Italy

## Abstract

*Objective*. Juvenile Idiopathic Arthritis (JIA) is the most common chronic pediatric rheumatic disease. It is recognized that only reliance on clinical signs of disease outcome is inadequate for understanding the impact of illness and its treatment on child's life and functioning. There is a need for a multidisciplinary and holistic approach to children with arthritis which considers both physical and emotional functioning. This study investigated the psychosocial functioning of children and adolescent with JIA and the disease-related changes in their family. *Methods*. The sample consisted of 33 hospitalized patients, aged 6–16 years. Both parents and the children were given a number of questionnaire to fill out. Clinical information was extracted from the interviews. *Results*. Self-reported psychological functioning (depression, anxiety, and behavior) was not different from the normal population; however significant psychological suffering was detected by the clinical interview. *Conclusions*. Children and adolescents with JIA do not show overt psychopathology by structured assessment; nevertheless a more clinically oriented holistic approach confirms JIA as a disrupting event causing relevant changes in the quality of life of the affected families.

## 1. Introduction

Juvenile Idiopathic arthritis (JIA) is a chronic inflammatology disease of unknown etiology, manifesting in children and adolescents, sometimes affecting internal organs [1]. If not appropriately treated, arthritis causes pain, limited joint motion, and anchylosis and results in significant disability.

This condition requires a complex and multidisciplinary approach which includes specialistic evaluations, pharmacological therapy, physical rehabilitation, and psycho-educational interventions addressing the children and their family needs [2, 3].

The family is necessarily a target of intervention in pediatric disabling conditions, both because of the crucial relationship of dependence in developing age and because of the impact of a chronic health condition affecting the child on the family setting [4].

In chronic rheumatic diseases, an effective approach to the psychological aspects of the disease should target the child in relation to his/her context and cannot neglect social aspects for a comprehensive understanding of the global experience of the disease.

Many authors underline the importance of providing appropriate environmental and family support to help the child adjust to the disease [5, 6]. The psychosocial outcome of patients with chronic rheumatic diseases is more influenced by family chronic difficulties than by the severity of the disease itself [7], and the sound social functioning of the parents facilitates child's coping with the disease [8]. Conversely the psychosocial well-being of the child can influence that of other family members [3]. Indeed, the frequent need for chronic drug treatment influences behavior and cognition.

The methodological difficulties in tackling this issue owing to the complexity of the variables at stake have been recognized by many authors [9, 10].

Also, we assessed the presence of psychological suffering and disease-related changes in the family and social contexts by using standardized and not standardized instruments.

## 2. Material and Methods

This study involved children and adolescents with JIA who were referred to our tertiary care Rehabilitation Unit for intensive rehabilitation. The illness was in active phase and all patients had JIA for at least six months.

The families gave their informed consent to participate in the study which was approved by the internal revision board.

Data about the family functioning were collected by interviews with parents during hospital stay which lasted from 7 to 15 days. Our sample consisted of 33 hospitalized patients, aged 6–16 years (mean age 10.5 ± 4.02), 23 females and 10 males.

The sample was divided into two age groups: the first (Group 1: children group) included 16 patients younger than 11 years (range 6–10 years), and the second (Group 2: adolescent group) included 17 patients older than 11 years (range 11–16 years).

### 2.1. Assessment Tools

Patients and their families received a psychological evaluation including both standardized tests and open interviews, performed by a psychologist trained in chronic disease.

 The standardized tests included the following

1. the Anxiety Checklist for Children and Adolescents [11] aged 7–15 years assessing environmental anxiety (EA), school-related anxiety (SA), and global anxiety (GA);
2. the Italian adaptation of the Children's Depression Inventory (CDI) [12] for children and adolescents aged 8–17 years assessing depressive symptoms such as mood disorders, self-esteem, and social behavior disorders, to make a diagnosis of psychopathological disturbance;
3. Achenbach's CBCL 6–18 yrs [13] evaluating parents' opinion on behavioral and social profile of their children. The first part of the CBCL investigates the quality of the child's participation in sports, house chores, and school activities, and at the quality of family and extrafamilial relations. Subscales of the second part for behavioral evaluation allow drawing a behavioral profile based on withdrawal, somatic complaints, anxiety/depression, social problems, thought problems, attention problems, delinquent behavior, and aggressive behavior. Furthermore, these subscales allow a bidimensional classification: the internalizing grouping taps intrapsychic problems, while the externalizing grouping taps conduct symptoms.

The need for qualitatively significant data that can be translated into ordinal data led us to organize interviews according to areas, with open-ended questions.

**Figure 1:**
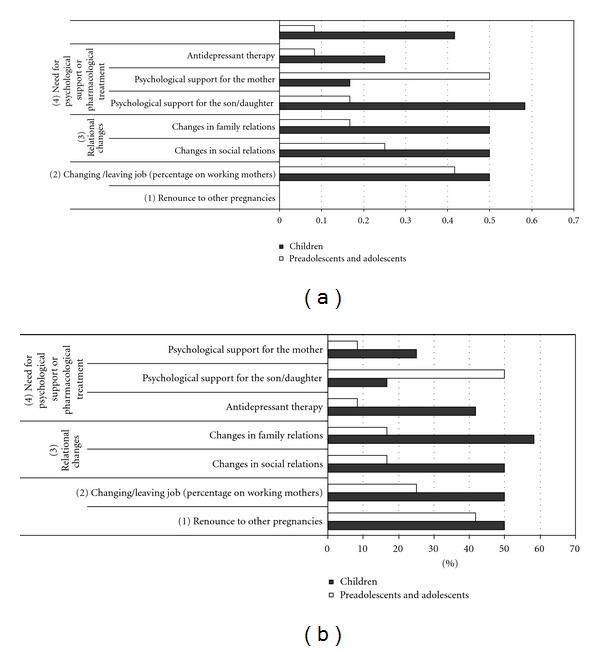
Changes in family context.

**Figure 2:**
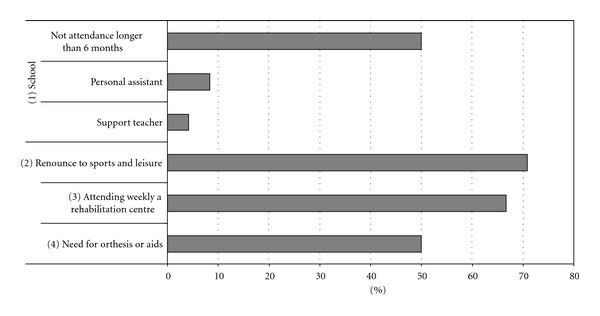
Quality of life.

**Figure 3:**
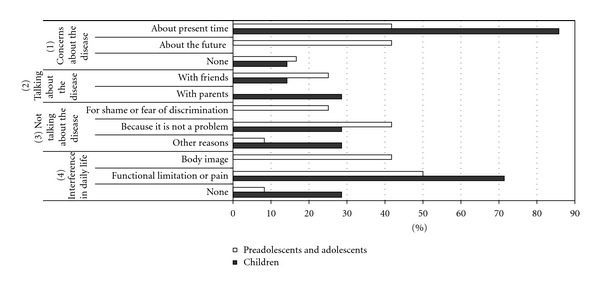
Experience of disease.

A coding system was produced based on descriptive categories which are reported in Figures 1, 2, and 3 *(changes in the family context, quality of life, disease experience*).

(1) Interviews with parents focused on the following
(i) collection of preliminary data: knowledge of the family (parents age, occupation, etc.), psychological development of the child before and after disease onset, disease, and relationship with the child (physical, emotional, and behavioral description of the child, relational and educational approaches);
(ii) disease and possible changes in the family context (see Figures 1(a) and 1(b)):
    (a) changes in family relations (problems with partner, relationships with other children) and social relations (reduced or changed relations),
    (b) need to change or leave job for the parents,
    (c) renounce to other pregnancies,
    (d) need for antidepressant therapy for the parents or need for psychological support for the parents or the son/daughter,
(iii) disease and quality of life of the child (see Figure 2):
    (a) school: attendance, need for certifications (support teacher, personal assistant),
    (b) renounce to sports and leisure,
    (c) treatment plans: attending weekly a rehabilitation centre,
    (d) need for orthesis or aids.
(2) Interviews with patients were aimed at exploring the impact of the disease on their emotional and relational dimensions and focused on the following (see Figure 3):
  - concerns about the present disease (pain, functional limitations, drug side effects, medical procedures) and possible future course (developing deformities, changes in body image, pregnancies);
  - possibility to speak about the disease with family members, friends, others, or, if not, why;
  - interference in daily living due to functional limitations or pain, or changes in body image.

## 3. Results

### 3.1. Standardized Tests

The Developmental Anxiety Scale and the CDI were administered to 9 children and 17 adolescents, and to 3 children and 17 adolescents, respectively. For both scales, age-related norms were applied.

On the Anxiety Scale the *z*-score range was between −1.71 and 0.04. No clinically relevant symptoms of anxiety were found in either age groups.

The mean of the Anxiety Scale scores for children was in the normal range but higher than that of adolescents (children: *μ*: −0.99 ± 0.49; adolescents: *μ*: −0.65 ± 0.54), without a statistically significant difference.

CDI scores did not reveal depression in either groups (children and adolescents); *z*-score range was between −1.71 and 0.21.

The CBCL administered to parents of 16 children and 17 adolescents showed the following results.

1. The social competence profile in both children (*T* score mean: 28.2 ± 8.9) and adolescents (*T* score mean: 36 ± 6.1) was below the normal range (*T* score normal range >37).
2. The behavioral profile resulted normal in both groups (children: *T* score mean: 51.5 ± 8.6; adolescents: *T* score mean: 52.8 ± 9.2, normal range: <63). In children, the *T* score mean of internalizing scores was 53.7 ± 7.8 and of externalizing scores was 47.9 ± 10.1. This difference was not statistically significant.

In adolescents, the mean *T* score of internalizing scores was 56.1 ± 11.4, externalizing scores 49.1 ± 5.2 (*P* < 0.02).

In conclusion, by using a standardized methodology, no psychopathology was found in either age group, but both groups showed a social competence profile that was below the mean, especially for children, and albeit values were within a normal range.

### 3.2. Interviews

As shown in Figure 1(a), a substantial share of mothers reported changes in family relations (37.5%) and social relations (33.3%).

Such changes were more prominent in children's families: changes in family relations (58.3%) and in social relations (50%) (Figure 1(b)).

Most of working mothers (75%) changed their job or left it altogether because of their child's disease; some of the interviewees (45.8%) gave up the idea of another pregnancy due to the child disease. Finally psychological support were sought from mothers (16.6%) and from other siblings (33.3%). One-fourth of mothers were taking antidepressants.

Regarding the quality of life of our patients (Figure 2), 50% of them experienced prolonged school absences due to the disease, 8.3% need a personal assistant at school, 70.8% gave up practicing sports and/or leisure activities, 66.6% attend Rehabilitation Centers on a weekly basis, and, lastly, 50% of them use orthesis and aids.

Figure 3 shows that 85.7% of patients younger than 11 years are mostly concerned about the current state of the disease. Patients older than 11 years show an even distribution of worries (41.6%) between the current state of the disease and the future.

The majority of interviewees do not mention their disease with other people (57.1% of children and 75% of adolescents) and state that this happens because the disease “is not a problem” (28.5% of children, 41.6% of adolescents), for shame of the disease or fear to be discriminated (25% of adolescents), or for other reasons (28.5% of children, 8.3% of adolescents).

Lastly, the majority of subjects in both age groups report disease interferences in his/her daily living (71.4% of children, 91.6% of adolescents); in the children group these interferences are referred only to functional limitations and pain, while in the adolescent group 41.6% say that the disease affects his/her body image.

**Figure 4:**
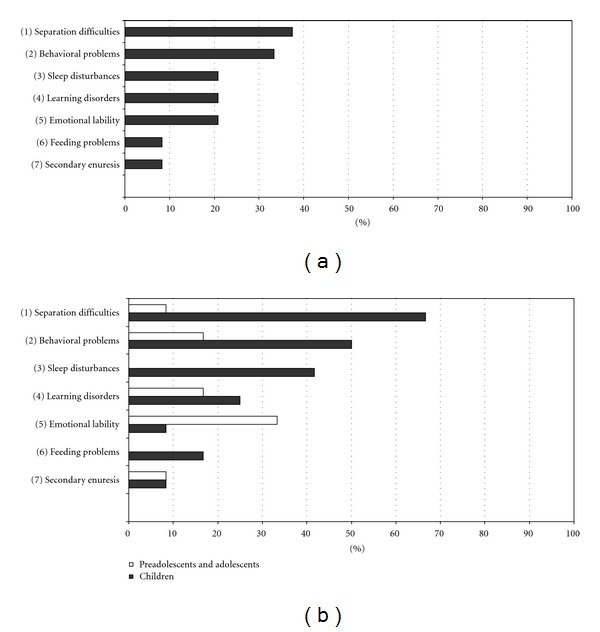
Psychological symptoms.

Regarding the presence of psychological suffering in our sample, interviews and clinical observation revealed symptoms of psychological unease in all the patients of group 1, and in 41.6% patients of group 2 (Figure 4(a)); more than one symptom could be present contemporarily in the same patient.

As shown in Figure 4(b), the distribution of symptoms was different in the two age groups: children had predominantly separation difficulties (66.6%) and oppositive provocatory disorder (50%), while adolescents demonstrated emotional lability (33.3%).

## 4. Discussion

Our work provides data on the psychosocial and familial unease in a sample of JIA pediatric patients.

The risk of emotional and behavioral difficulties experienced by children and adolescents with JIA has been assessed in previous studies. Most of them have examined parents or patients by standardized ratings and failed to identify in a relevant percentage of patients specific behavioral or emotional symptoms. Studies including observational formats requiring psychiatrist evaluation reported higher level of psychological symptoms in children with recent onset of rheumatic disease [14].

It is not easy to find a direct correlation between chronic rheumatic diseases and child psychopathology [6, 15], unless several particular aspects are considered such as extent of physical pain [9], degree of disability in daily activities [16], adolescent age [2], and quality of family support [5, 17].

In our experience the choice of a mixed approach (structured measures and not-structured tests), taking into account the emotional and cognitive level of the patient, allowed the detection of psychological suffering. This knowledge is crucial in order to understand the emotional impact of the disease on patients and their families and to provide appropriate help.

The importance of using a mixed evaluation methodology is suggested by the following aspects: length of the disease (did the patient adjust to the disease or does he/she not show to be suffering?), measures ceiling or floor effect (standardized checklists apparently do not detect mild adjustment difficulties; is it easier for patients to “lie”?), emotional responses that cannot be empirically verified (denial, coping styles), and specificity of age groups (difficulties in comparing different styles and expression abilities).

Furthermore exclusive reliance on structured checklists may lead to underestimation of anxiety and depression in that dysthymic symptoms such as loss of appetite, sleep disturbances, and lack of energy may be attributed to JIA.

According with previous reports [6, 15], in our study we did not find any pathological data as far as anxiety and/or depression.

However, a large part of our sample (100% in group 1, 41.6% in group 2) showed psychological unease as diagnosed by interviews, clinical observation, and collection of medical data (see Figure 4(a)).

Seven out of 33 patients (21.2%) were referred to a psychologist for treatment, and this is clinically relevant.

As already reported in other studies [18], the social competence profile in both children and adolescents was below the normal range and the assessment of patient's quality of life confirmed this finding (Figure 2). The severity of the disease forced our patients to prolonged school absences; in cases of early diagnosis they could not attend nursery school, had to give up playing/leisure activities, and motor activities and needed to attend a Rehabilitation Center on a regular basis.

This is quite relevant, especially when considering that, at this age, physical activity is one of the main means of communication and play with peers. Indeed, there is some parallelism between the high rate of school absences in children with JIA and repeated absences from work as well as withdrawal in affected adults [9]. In children too withdrawal due to a reduced school attendance may be equally disabling.

Interestingly, children were more concerned than adolescent about pain and functional deficits, and this underlines the impact that reduced physical activity and play may have on the child's well-being.

Adolescents concerns about the future are pertinent to prognosis, deformations, and feared changes in the physical aspect: their disease perception is more detached from daily life. The attention they pay to deformations and their body image is a measure of the meanings they attribute to the disease. Besides, they are more aware of the disease-related stressful events and associated fantasies [2].

Despite the frequency of these concerns, the majority of older patients do not talk about the disease either with parents or with friends. Children who do not talk about it show denial (“it's not a problem”), and adolescents are ashamed and fear discrimination. In line with these observations, adolescents show significantly more internalizing than externalizing symptoms.

This is probably related to a more developed ability to convey how they feel about it, and to the integration of emotional and cognitive aspects in their personality structure.

With regard to CBCL scores, some authors underline how environmental expectations are influenced by times of relative well-being rather than difficult times [3] and that the mothers' judgments about functional abilities are influenced by the presence/absence of pain. In the understanding of our CBCL results, we then have to consider that in our patients a pharmacological control of pain was achieved and may have affected the mother's perception of the disease.

Considering quality of life of the families, we could identified some “objective” and “subjective” indicators of family unease. “Objective” indicators concern a reduction in extrafamilial activities (work, relations with neighbors, spare time, etc.), while “subjective” indicators concern satisfaction with couple relationship and psychological well-being.

Accordingly, the data we collected from interviews showed that JIA is a disrupting event causing relevant changes in the quality of life of the families for both the above-mentioned indicators. This is clearly confirmed by the large proportion of working mothers giving up their job, by the substantial share of those reporting changes in their couple relationship and/or relationship with the other siblings, or suffering changes in social relations.

This last finding encourages reflection on the possible meaning of the disease in terms of loss, disrupting narcissistic attitudes (loss of representation of the child as healthy) and parenting role. Similar findings of increased risk of psychosocial stress, with higher percentage of psychological symptoms as compared with a control group, have been previously reported in mothers of JIA patients [19].

From a resarch standpoint the present work suffer because of the small cohort size and the lack of a control group, however it is relevant if we consider that as a result of this investigation, 29.1% of boys and 37.5% of mothers were sent to the psychologist.

In conclusion, our study shows the need for a clinical multi-informant approach to achieve a deeper understanding of how the patients with JIA and their families experience the disease. According to new ICF classification [20], the concept of health condition is the result of dynamic interaction between physical, mental, social, and environmental factors. Therefore, the pervasive albeit subtle effect of the condition on multiple facets of child and family functioning calls for a global approach to care and a structured and multidisciplinary working methodology.
